# 3D seismic evidence of buried iceberg ploughmarks from the mid-Norwegian continental margin reveals largely persistent North Atlantic Current through the Quaternary

**DOI:** 10.1016/j.margeo.2017.11.016

**Published:** 2018-05-01

**Authors:** A. Montelli, J.A. Dowdeswell, D. Ottesen, S.E. Johansen

**Affiliations:** a Scott Polar Research Institute, University of Cambridge, Cambridge CB2 1ER, UK; b Geological Survey of Norway, Trondheim N-7491, Norway; c Department of Petroleum Engineering and Applied Geophysics, Norwegian University of Science and Technology, Trondheim N-7031, Norway

**Keywords:** Icebergs, Ploughmarks, Ice stream, Norwegian Sea, North Atlantic Current, Fennoscandian Ice Sheet, Seismic stratigraphy, Marine geology, Palaeo-glaciology, Glacial geomorphology

## Abstract

Over 7500 buried linear and curvilinear depressions interpreted as iceberg ploughmarks were identified within the Quaternary Naust Formation from an extensive three-dimensional seismic dataset that covers ~ 40,000 km^2^ of the mid-Norwegian continental margin. The morphology and net orientation of ploughmarks were mapped and analysed. These features are up to 28 km long, 700 m wide and are incised up to 31 m deep. On average, ploughmarks are incised 5 m deep, with median width of 185 m and median lengths ranging from 1.2 to 2.7 km for individual palaeo-surfaces. Width to depth ratio ranges from 8:1 to 400:1 and is on average 36:1. The presence of ploughmarks buried deeply within some palaeo-slope surfaces implies the occasional presence of very large icebergs since the middle Quaternary, suggesting that thick ice-sheet margins with fast-flowing ice streams were present in order to calve icebergs of such dimensions into the Norwegian Sea. The wide geographical distribution of ploughmarks suggests unrestricted iceberg drift and an open Norwegian Sea during the periods of iceberg calving since the early Quaternary. Ploughmark trajectory analysis demonstrates that the ocean current circulation, now dominated by the northeasterly flowing Norwegian Atlantic Current (NwAC), has largely persisted throughout the Quaternary. Despite the overall strikingly consistent pattern of iceberg drift, ploughmark mapping also shows evidence for short-lived NwAC reductions possibly related to major phases of iceberg discharge and/or meltwater pulses from the Fennoscandian Ice Sheet during the middle and late Quaternary.

## Highlights

• Over 7500 buried iceberg ploughmarks were identified within the Quaternary Naust Formation
• Statistical analysis provides detailed morphometric information on buried ploughmarks on the mid-Norwegian margin
• The wide geographical distribution of ploughmarks suggests unrestricted iceberg drift and an open Norwegian Sea since the early Quaternary
• Ploughmark trajectory analysis demonstrates that the Norwegian Atlantic Current (NwAC) has largely persisted throughout the Quaternary
• There is evidence for short-lived NwAC reductions possibly related to major phases of iceberg discharge and/or meltwater pulses

## 1 Introduction

Icebergs affect the oceanography and geological record of continental margins in several ways. First, they represent a dominant source of present-day mass loss from ice sheets and can make a significant contribution to the freshwater balance of the oceans (Broecker and Denton, 1989, Seidov et al., 1996, Stokes et al., 2005, Depoorter et al., 2013, Rignot et al., 2013). Thus, periods with large fluxes of icebergs can indicate episodes of rapid disintegration and mass loss from ice sheets and/or ice shelves (*e*.*g*., Green et al., 2010, Weber et al., 2014, Wise et al., 2017). Secondly, ice rafting is a key mechanism for transporting sediment into distal parts of deep-ocean basins, often several thousands of kilometres away from iceberg sources (*e*.*g*., Heinrich, 1988, Broecker et al., 1992, Hemming, 2004, Kuijpers et al., 2007, Hill et al., 2008). Finally, when in contact with the seafloor, the ploughing activity of iceberg keels shapes the morphology of the substrate affecting, for example, local biodiversity and any engineering structures that are present (Dowdeswell et al., 1993, Syvitski et al., 1996, Gutt et al., 1996, O'Brien et al., 1997, Laudien et al., 2007).

Indicators of past iceberg activity found within the sedimentary and geomorphological records of continental margins take several forms. Sedimentological evidence includes the abundance, grain-size distribution and lithology of glacigenic ice-rafted debris (IRD) commonly found in sediment cores from high-latitude seas (Heinrich, 1988, Zachos et al., 1992, Bond et al., 1992, Alley and MacAyeal, 1994, Dowdeswell et al., 1995, Dowdeswell et al., 1999, Ó Cofaigh et al., 2002). Geomorphological indicators, including iceberg ploughmarks and grounding pits, are probably the most characteristic of glacimarine landforms that have been observed widely on continental shelves and upper slopes, at depths of up to 1 km and equatorward as far as the subtropical North Atlantic and Chatham Rise off New Zealand (*e*.*g*., Belderson et al., 1973, Dowdeswell et al., 1993, Polyak et al., 2001, Hill et al., 2008, Hill and Condron, 2014, Dowdeswell et al., 2016, Stewart, 2016).

Iceberg ploughmarks represent linear to curvilinear scours incised into seabed sediments. Ploughmarks form as a result of iceberg keels coming into contact with the seafloor after calving from the marine termini of glaciers and ice sheets (Dowdeswell and Bamber, 2007). Such contact can happen either upon their drift into shallower areas and/or roll-over resulting from local calving and ablation (Woodworth-Lynas et al., 1991). As iceberg calve from their parent ice masses, they subsequently drift into the adjacent oceans, ploughing the seafloor and thus recording the trajectories of their past drift (Dowdeswell and Bamber, 2007). The direction of iceberg transport is determined by the sum of forces acting on their surface areas, with major control exerted by ocean currents given that about 90% of their deep keels are below the sea-surface; ploughmarks are therefore a useful proxy for past ocean circulation (Tchernia and Jeannin, 1980, Todd et al., 1988, Bigg et al., 1996, Aoki, 2003, Kristoffersen et al., 2004, Schodlok et al., 2006, Hill et al., 2008, Newton et al., 2016).

Observations of iceberg ploughmarks can be obtained using multibeam swath-bathymetric, side-scan sonar or three-dimensional (3D) seismic records, each of which allows the seafloor (and buried former seafloors in the case of 3D seismics) to be imaged at the necessary resolution (*e*.*g*., Woodworth-Lynas et al., 1991, Andreassen et al., 2007, Dowdeswell and Ottesen, 2013, Jakobsson, 2016). These methods enable the identification of multiple characteristics that are unique to iceberg ploughmarks, such as the presence of lateral berms, the often chaotic pattern of their spatial distribution, grounding pits and surcharges of sediment at the terminations of grooves (Sacchetti et al., 2012). The dimensions of ploughmarks are spatially variable, with features that are typically tens to hundreds of metres wide and a few to tens of metres deep (*e*.*g*. Dowdeswell et al., 1993).

In the North Atlantic, numerous iceberg ploughmarks are present on the seafloor of modern mid- and high-latitude continental shelves, recording the history of iceberg drift predominately during the most recent glacial cycle (*e*.*g*., Ó Cofaigh et al., 2002, Hill et al., 2008, Metz et al., 2008). Where 3D seismic datasets are available, ploughmarks have also been found on seismic palaeo-surfaces that represent the former seafloor, buried up to almost 1 km deep within the Quaternary sediments (Jansen and Sjøholm, 1991, Eidvin et al., 2000, Andreassen et al., 2008, Dowdeswell and Ottesen, 2013, Bjarnadottir et al., 2016, Newton et al., 2016). Identification of such buried ploughmarks provides an important record of the temporal and spatial variability of iceberg production and the oceanographic evolution of the adjacent continental margin through multiple glacial-interglacial cycles (Dowdeswell et al., 1993, Newton et al., 2016).

**Fig. 1:**
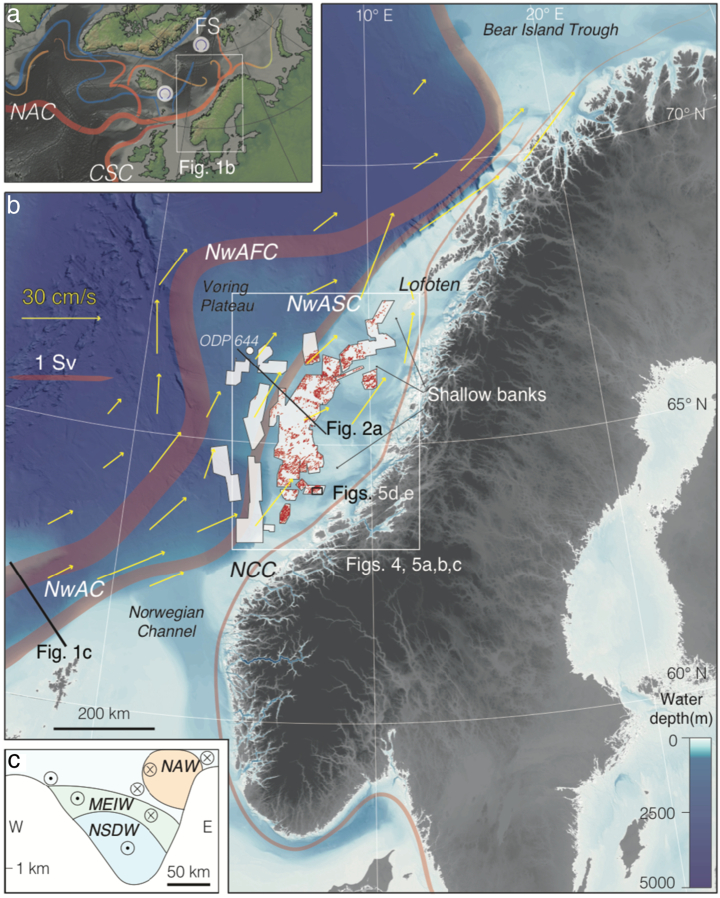
The Norwegian continental margin. a. Large-scale ocean-surface circulation in the North Atlantic. NAC – North Atlantic Current. CSC – Continental Slope Current. FS – Fram Strait. Circles represent sources of deep water (simplified from Hansen and Østerhus, 2000). b. Norwegian continental margin morphology showing shallow banks adjacent to major deep glacial cross-shelf troughs incised into the shelf and branches of ocean-surface circulation in the Norwegian Sea (modified from Hansen and Østerhus, 2000) in the study area (white box, Fig. 4, Fig. 5a,b,c). Red, thick semi-transparent lines show the major prevailing surface currents (NwAC – Norwegian Atlantic Current and its major branches: NwAFC – Norwegian Atlantic Front Current and NwASC - Norwegian Atlantic Slope Current). NCC - Norwegian Coastal Current. Thicknesses of red lines are roughly proportional to mass fluxes of respective currents, in Sv (from Gascard et al., 2004); 1 Sv = 10^6^ m^3^ s^− 1^. Yellow arrowed lines represent mean velocity vectors computed from combined drogued and wind-corrected undrogued drifter observations from August 1991 to December 1994 (Poulain et al., 1996). Small red line segments show iceberg ploughmarks preserved on the modern seafloor and interpreted from the available 3D seismic cubes. Thin black line shows the 2D dip seismic line GMNR94-106. 3D seismic data coverage of the study area is represented by semi-transparent white areas. Thick black line shows the transect between Faeroe and Shetland Islands (Fig. 1c). c. Schematic diagram of the typical distribution of the main water masses with arrows indicating general flow through the section. Water masses: NAW - North Atlantic Water, MEIW Modified East Icelandic Water, NSDW Norwegian Sea Deep Water. Modified from Gould et al. (1985) and Hansen and Østerhus (2000). (For interpretation of the references to colour in this figure legend, the reader is referred to the web version of this article.)

This paper focuses on ~ 7500 ploughmarks identified within the 2.6 Myr sedimentary record contained in the glacigenic Quaternary Naust Formation using extensive (~ 40,000 km^2^) 3D seismic records from the mid-Norwegian continental shelf and slope (Fig. 1). We investigate the morphology, dimensions and spatial patterns of iceberg ploughmarks along this 500-km long continental margin and discuss the palaeo-environmental implications of these observations through multiple glacial-interglacial cycles.

## 2 Background

### 2.1 Continental margin morphology

The modern morphology of the mid-Norwegian continental margin (64–68°N) is characterized by an outward-bulging shelf configuration and several cross-shelf troughs (Fig. 1b) produced by the erosional activity of palaeo-ice streams that drained the Fennoscandian Ice Sheet (FIS) in the middle and late Quaternary. Shelf width is as narrow as 50 km in the southern (~ 62–63°N) and northern (~ 68–69°N) parts of the margin and up to 250 km wide in the central area (~ 65–66°N) (Fig. 1b). The slope gradients are gentle (~ 1°) on the Vøring Plateau (in the central sector of the study area) and steeper (up to 5°) in areas adjacent to narrow-shelf regions. Shelf depths range from ~ 150 m on shallow banks to ~ 550 m in the deep glacial cross-shelf troughs (Dahlgren et al., 2002, Ottesen et al., 2005). The seafloor topography in the study area provides an important control on the regional current system (*e*.*g*., *via* intensification of current speeds due to an increase in slope gradients) (*e*.*g*., Orvik and Niiler, 2002, Bryn et al., 2005).

### 2.2 Chronostratigraphic framework

**Fig. 2:**
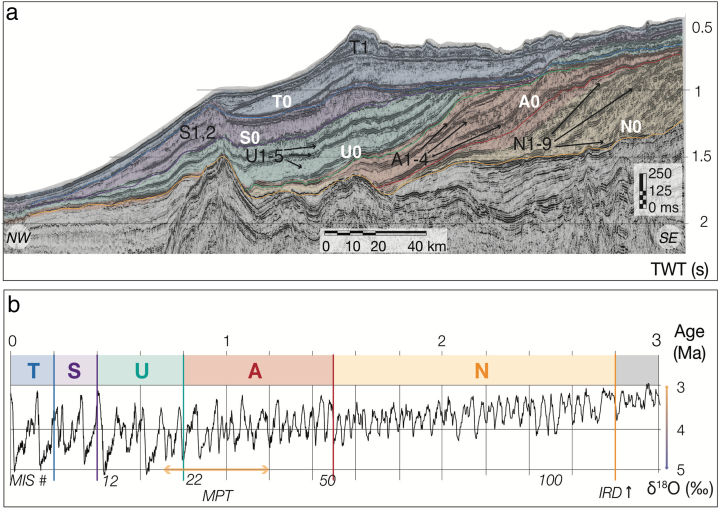
Naust Formation chronostratigraphy. a. Dip seismic profile GMNR94–106 showing Naust sequences (located in Fig. 1b). N0 represents the basal Naust surface. b. Approximate chronology of the Naust Formation evolution (from Rise et al., 2010) with the δ^18^O marine-isotope time series as a global ice volume proxy (from Lisiecki and Raymo, 2005). Major events are annotated to the left: IRD – early Quaternary increase in ice-rafted debris (Jansen and Sjøholm, 1991). MPT – the middle-Pleistocene Transition (~ 1.2–0.75 Ma) (Clark et al., 2006). Modified from Montelli et al. (2017).

During the Quaternary, uplift onshore and the onset of glaciation led to the deposition of ~ 100,000 km^3^ of mainly glacigenic Naust Formation on the mid-Norwegian continental margin (*e*.*g*., Rise et al., 2005, Dowdeswell et al., 2010a, Ottesen et al., 2012). The oldest part of the Naust Formation comprises strongly progradational, gently dipping units (Fig. 2a) (*e*.*g*., Dahlgren et al., 2005, Ottesen et al., 2009). Mainly flat-lying units of the Naust Formation deposited in the Middle-Late Quaternary mark the architectural shift of the margin (*e*.*g*., Ottesen et al., 2009). Due to the dipping character of the palaeo-shelf surfaces, the buried outer shelves are often well-preserved, allowing examination of the ploughmark record through the Quaternary.

According to previous studies based of the ice-rafted debris (IRD) recovered from ODP wells 642–644 in the Vøring Plateau (Fig. 1), glaciations occurred on the Norwegian coast ~ 2.8 Ma (*e*.*g*., Jansen and Sjøholm, 1991). While the Early Quaternary Naust units (*i*.*e*., ~ 2.7–0.8 Ma) remain tentatively dated due to the lack of well-dated core material (*e*.*g*., Rise et al., 2005, Ottesen et al., 2009), most of the Middle-Late Quaternary Naust chronostratigraphy (*i*.*e*., ~ 0.5 Ma to present) has been relatively well-constrained using ODP 644 sediment cores (*e*.*g*., Haflidason et al., 2001, Dahlgren et al., 2002).

### 2.3 Oceanographic setting

At present, the surface-current system in the North Atlantic (Fig. 1b) is dominated by warm and saline Atlantic Water flowing north-eastward into the Nordic Seas in the wind-driven, topographically steered Norwegian Atlantic Current (NwAC) that constitutes the northern end of the North Atlantic Current (NAC) (Hansen and Østerhus, 2000, Orvik and Niiler, 2002). The NwAC is composed of two major branches (*e*.*g*., Poulain et al., 1996, Orvik and Niiler, 2002, Søiland et al., 2008) referred to as the Norwegian Atlantic Slope Current (NwASC) that flows along the shelf-break (Skagseth and Orvik, 2002) and the Norwegian Atlantic Front Current (NwAFC) which flows in deeper water (Mork and Skagseth, 2010, Hansen et al., 2011). Average current velocities of the NwAC are in the range of 0.2–0.4 cm s^− 1^ (Poulain et al., 1996, Ersdal, 2001). The other major current in the Norwegian Sea is the Norwegian Coastal Current (NCC) that flows along the Norwegian coast until its amalgamation with NwASC in the Lofoten area at ~ 68° N (Fig. 1b). Overall, the northeasterly direction of the modern surface-current system on the mid-Norwegian margin ranges between 0° and 60° (Fig. 1b).

Warm, saline surface waters of the NwAC gradually cool, flowing into the Fram Strait area, where they eventually sink and outflow (Fig. 1a) *via* the cold East Greenland Current, forming one of the major branches of the Atlantic Meridional Overturning Circulation that constitutes a significant portion of North Atlantic Deep Water (Broecker et al., 1998, Hansen and Østerhus, 2000, Davies et al., 2001, Hohbein et al., 2012). Thus, the Norwegian margin is an important region that transmits crucial links between deep and shallow circulation in the North Atlantic (Schmitz and McCartney, 1993, Dickson and Brown, 1994, Seidov and Maslin, 1999, Hansen and Østerhus, 2000, Mokeddem and McManus, 2016). Therefore, buried glacier-influenced surfaces along the mid-Norwegian margin are well situated to record the spatial variability of the northern limit of the NAC through the Quaternary.

### 2.4 Glaciological setting

Studies of sediment cores and 3D seismic datasets have shown that the mid-Norwegian margin was influenced by repeated expansions of the calving FIS margin since the earliest Quaternary (Jansen and Sjøholm, 1991, Dahlgren et al., 2002, Rise et al., 2005, Montelli et al., 2017). Fast-flowing ice streams, which drain large ice-sheet interior basins, represent the major source of iceberg production both today and in past ice sheets (*e*.*g*., Lien, 1983, Bamber et al., 2000, Ottesen et al., 2005, Rignot and Kanagaratnam, 2006). Such ice streams appear to have developed mainly since the middle Quaternary of the FIS, with their major growth episode occurring around the end of the mid-Pleistocene Transition (~ 1.2–0.7 Ma) (*e*.*g*., Siegert and Dowdeswell, 2002, Clark et al., 2006, Montelli et al., 2017, Reinardy et al., 2017). During the Last Glacial Maximum (LGM) about 20 kyr ago, the western margin of the FIS was drained by several palaeo-ice streams, many of which eroded major glacial troughs along the mid-Norwegian shelf (*e*.*g*. Rise et al., 2005, Ottesen et al., 2005).

Previous ice-sheet numerical modelling experiments suggested that the thickness of the marine margin of the FIS on the mid-Norwegian shelf may have been up to 1000 m at the modern coastline during the LGM (*e*.*g*., Peltier, 1994, Dowdeswell and Siegert, 1999). At the marine margins of the FIS, iceberg production was focused at the mouths of cross-shelf troughs, in which fast-flowing ice streams were located. The largest of these ice streams was the Norwegian Channel Ice Stream to the south of the study area (Fig. 1), which, according to the numerical models (*e*.*g*., Siegert and Dowdeswell, 2002), produced icebergs at a rate of up to 35 km^3^ yr^− 1^ between 16,000 and 14,500 kyr ago. Modelled iceberg flux during the last, Weichselian deglaciation for the entire FIS peaked between 15 and 12.5 kyr ago, reaching rates of up to 2000 km^3^ yr^− 1^ (Siegert et al., 1999, Siegert and Dowdeswell, 2002).

## 3 Methods

### 3.1 Seismic dataset and interpretation

This study uses multiple and partly overlapping 3D seismic cubes from the Schlumberger Petrel® Ready Database covering ~ 40,000 km^2^ of the mid-Norwegian margin. The acquisition parameters of separate seismic surveys generally include dual sources with 25–50 m separation and 2 to 6 streamers, each of 3000–4000 m length, towed at depths of 5–10 m. The shot-point interval was 25 or 50 m and the sampling rate for all surveys was 2–4 ms. Standard seismic imaging workflow and software were used by Schlumberger Geco and Petroleum Geo Services to process the datasets. All the data used in this study were in two-way travel time (TWT). Assuming a dominant frequency of ~ 50 Hz and a sound velocity of ~ 2000 m/s (Ottesen et al., 2009), the vertical resolution of the dataset is up to 10 m.

**Fig. 3:**
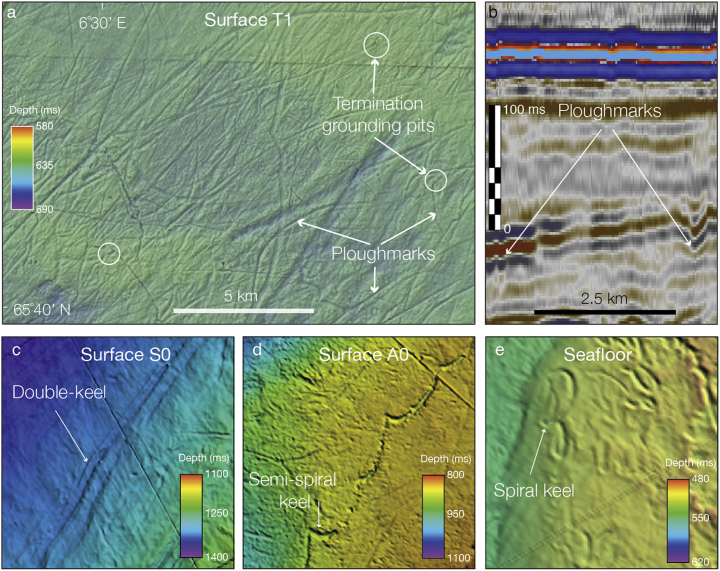
Examples of buried linear and curvilinear incisions interpreted as iceberg ploughmarks in the study area. a. Structure amplitude map of ploughed palaeo-surface T1 (3D seismic cube ST9301). b. Part of seismic cube ST9301 (Inline 2819) showing an example of cross-sectional image of buried ploughmark on a 2D seismic line. c. Linear double-keeled ploughmarks produced by large tabular icebergs (found on the palaeo-slope surface within the Naust S sequence). d. Curvilinear semi-spiral iceberg ploughmark present on the early – middle Quaternary palaeo-surface A0 (occurrence of such features within the Naust Formation is rare compared to regular linear cross-cutting keels) e. Example of spiral-keeled ploughmarks found on the modern seafloor (occurrence of such features is relatively rare).

The auto-tracking method in Schlumberger Petrel® software was used to produce high-resolution individual amplitude maps (up to 12.5 m spaced grids) for 27 individual erosional unconformities defined in Montelli et al. (2017) for the Naust Formation (Fig. 2). The Naust stratigraphy is from Ottesen et al. (2009) and Montelli et al. (2017), and provides a regional-scale framework for more detailed palaeo-shelf and -slope morphological interpretations in this paper (Fig. 2a). In this study, the first letter of each palaeo-surface name stands for the sequence within the Naust Formation which contains that surface. Numbers represent the chronostratigraphic order of palaeo-surfaces within respective units, from oldest to youngest (Fig. 2a). The interpreted palaeo-surfaces were then converted into the raster grids (re-sampled to 25–50 m resolution) used in this analysis (Fig. 3). The raster grids of interpreted surfaces were visualized in ArcGIS® as hill-shaded images that represent the basis for extensive mapping of seafloor and buried ploughmarks.

### 3.2 Ploughmark mapping and statistics

**Fig. 4:**
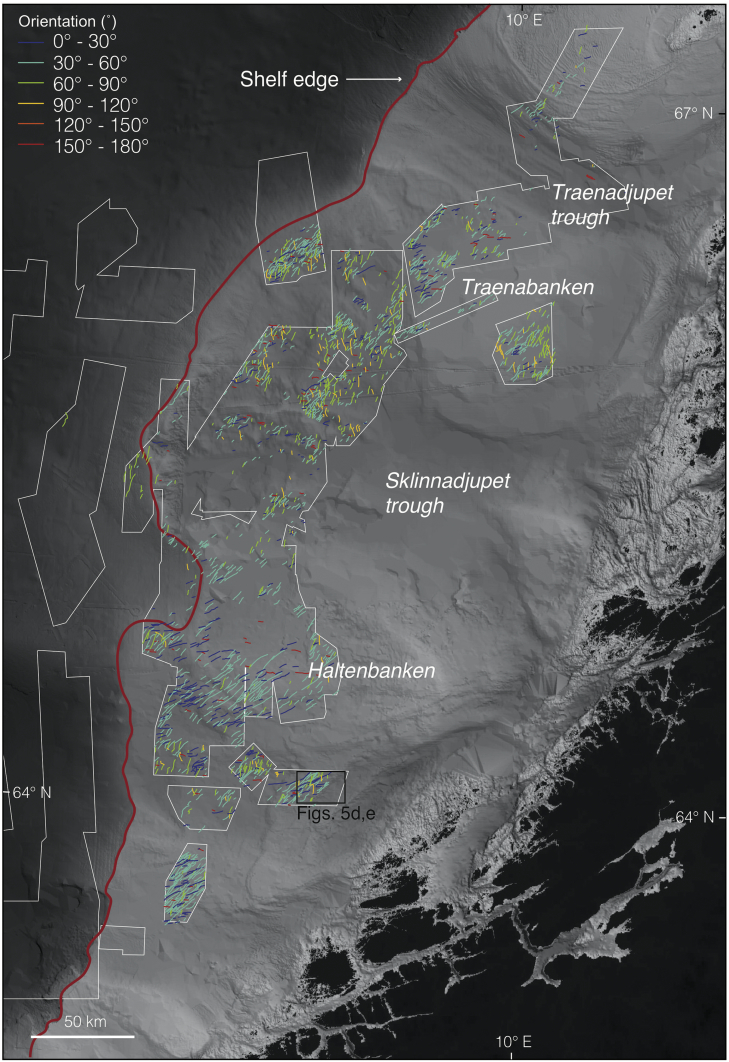
Ploughmarks mapped on the modern seafloor surface of the mid-Norwegian shelf and uppermost slope from the available 3D seismic dataset (located in Fig. 1b). Each ploughmark is coloured according to its net orientation. White boxes outline the 3D seismic dataset used in the study area. Red line shows the modern shelf break. (For interpretation of the references to colour in this figure legend, the reader is referred to the web version of this article.)

**Fig. 5:**
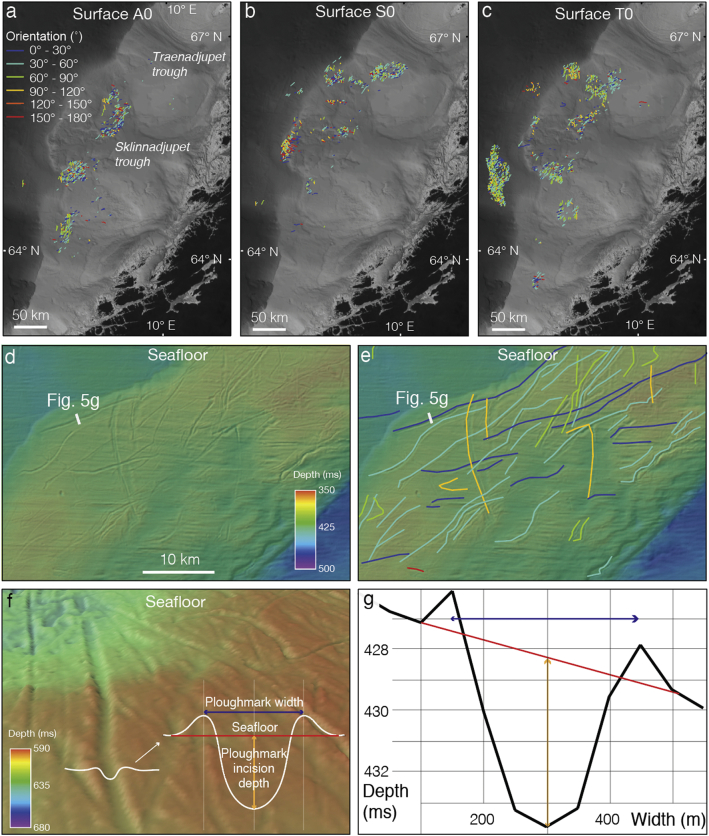
Mapping and morphometric analysis of buried iceberg ploughmarks on the mid-Norwegian continental margin. a,b,c. Examples of ploughmarks mapped on palaeo-surfaces A0, S0, T0 from the available 3D seismic dataset. Ploughmarks are coloured according to their orientation. d. Zoomed-in section of the study area showing the cross-cutting character of linear and curvilinear incisions on the modern seafloor. e. Interpreted ploughmarks of Fig. 5d coloured according to their orientation. f. Three-dimensional view of a ploughmark and a black line showing its cross-sectional profile and idealised sketch showing definition of main ploughmark dimensions used in this study. g. Application of the measurements defined in Fig. 5f based on a real ploughmark from the modern seafloor surface shown in Fig. 5d, e.

The exported raster grids were used to trace and digitise each individual ploughmark (Fig. 4, Fig. 5a,b,c) in ArcMap® software. Due to their relatively small incision depth (usually no more than a few metres), ploughmarks typically represent very subtle indentations on 2D seismic data (Fig. 3b) (*e*.*g*., Batchelor et al., 2013). However, when mapped in three dimensions, these depressions form a systematic pattern (Fig. 3, Fig. 4) that can easily be traced and mapped on surfaces buried under a kilometre or so of overlying sediments (*e*.*g*., Dowdeswell and Ottesen, 2013, Newton et al., 2016). Identification of ploughmarks was based on their dimensions and the presence of unique morphological characteristics, including their cross-cutting linear and curvilinear character, chaotic distribution and the presence of lateral berms (*e*.*g*., Belderson et al., 1973, Woodworth-Lynas et al., 1985, Ó Cofaigh et al., 2002, Dowdeswell et al., 2007, Dowdeswell et al., 2010b, Sacchetti et al., 2012, Dowdeswell and Ottesen, 2013).

The total length and net orientation (within the angular range of 0°–180°) of all mapped ploughmarks was calculated automatically in ArcGIS®. The orientation is defined as an angle between the horizontal axis (*i*.*e*., latitude) and the line connecting start and end points of ploughmark polyline. The values of the orientation angle increase counterclockwise, starting at 0° in the east and going through 90° when the major axis is vertical (*e*.*g*., Fig. 5). Unusual, spiral-shaped ploughmarks have been excluded from the orientation analysis.

Extraction of the width and depth of interpreted ploughmarks on each palaeo-shelf surface was completed along cross section profiles based on randomly sampled ploughmark populations, where each population represents respective palaeosurface. Locations of cross section profiles were chosen approximately at the middle of each ploughmark. Sample size (*n*) for each population (*i*.*e*., each palaeo-surface) was calculated separately with a 7.5% margin of error and 95% confidence level using the formula for finite populations:

$$n=\frac{m}{1+\frac{m-1}{N}}$$

where *n* is the sample size with the finite population correction, *N* is the total population size and *m* is the sample size without considering the finite population correction factor calculated using the formula:

$$m=\frac{z^{2}p\left(1-p\right)}{e^{2}}$$

where *z*^2^is the confidence level parameter, representing abscissa of the normal curve that cuts off an area α at the tails (1 – α) equals the desired confidence level, *e*.*g*., *z*^2^ of 1.96 for confidence level of 95%), *e* is the desired level of precision (margin of error), *p* is the estimated proportion of an attribute that is present in the population (value of 0.5 assuming maximum variability) (Israel, 1992).

Based on this analysis a subset of 1997 ploughmarks were sampled for further width and depth analysis. For each interpreted ploughmark from the random subsets, its dimensions were systematically measured on cross-sectional profiles (Figs. 5e,f) using the width and depth definitions outlined in Figs. 5e,f,g. Final visualisation and calculation of the main statistical parameters was carried out in Matlab®.

## 4 Results

Analysis of high-resolution 3D seismic data in the study area reveals that the modern- and palaeo-seafloor surfaces of the mid-Norwegian shelf and upper slope are incised by over 7500 elongate linear, curvilinear and, in rare cases, spiral-shaped depressions (Fig. 3). Some of these features terminate in semi-circular pits bounded by small push-up ridges sometimes known as surcharges (Fig. 3a). The overall distribution pattern of these depressions is irregular, with cross-cutting relationships and abrupt orientation changes. The character and dimensions of the features are very similar to those found by previous side-scan sonar, swath-bathymetric and 3D seismic studies from other high-latitude margins, where the seafloor sediments were heavily ploughed by drifting icebergs (*e*.*g*. Woodworth-Lynas et al., 1985, Dowdeswell et al., 1993, Syvitski et al., 2001, Hill et al., 2008, Dowdeswell and Ottesen, 2013, Newton et al., 2016). These features are, therefore, interpreted as iceberg ploughmarks.

**Fig. 6:**
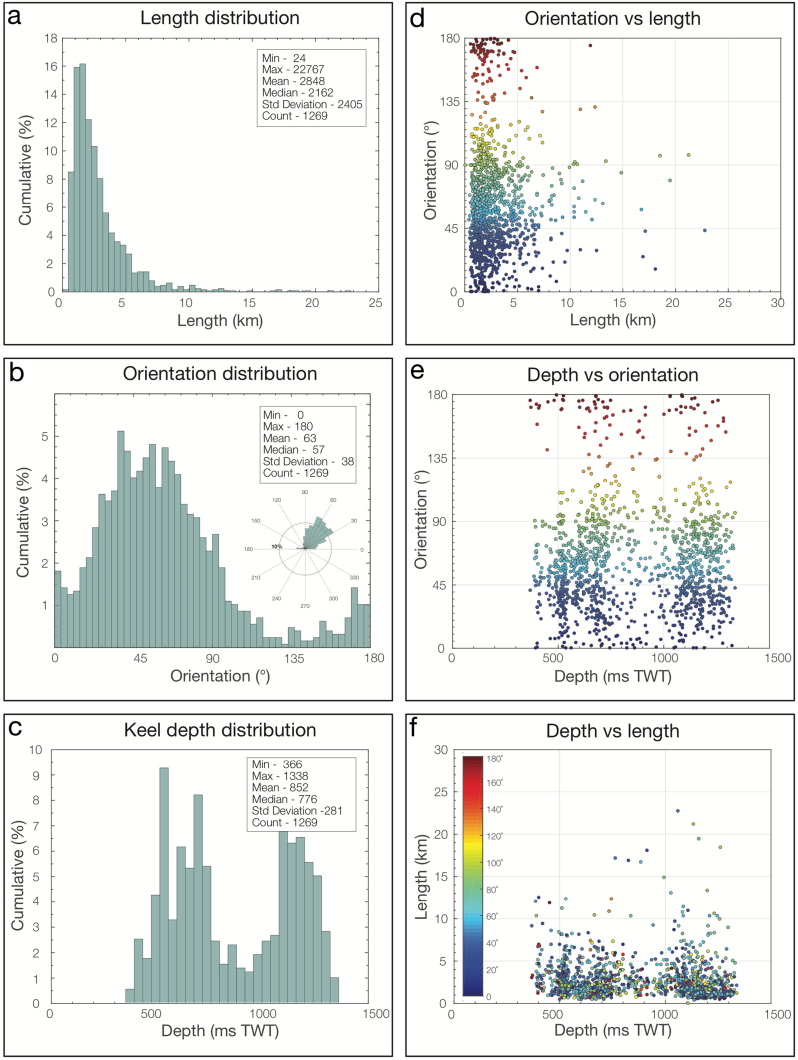
Visualized ploughmark statistics. Example of frequency distribution of the length (a), orientation (b), keel depth below the present seafloor (c) of iceberg ploughmarks buried within the palaeo-surface T0 (Base of the Sequence T) and scatter plots of orientation *vs* length (d), keel depth below seafloor *vs* orientation (e) and keel depth below seafloor *vs* length (f). Data points are coloured according to the orientation.

Ploughmarks buried within the Naust Formation exhibit a range of morphologies and are present in various parts of the mid-Norwegian margin at different depths, from shallow banks on the shelf to relatively deep parts of the slope. Fewer ploughmarks were found within the old units of Sequence N as opposed to the younger Naust sequences (Table 1). A synthesis of ploughmark dimensions and orientations for each interpreted palaeo-surface is presented in Table 1 and an example of scatterplots and normalized frequency histograms (for the base of the Naust Sequence T) is shown in Fig. 6.

**Table 1:** Summary of key statistics for different parameters of all interpreted ploughmarks on the mid-Norwegian margin, including length, orientation, keel depth below the seafloor, keel incision depth, width, and width/depth ratio summarized for each palaeo-surface.

Surface name		SF	T1	T0	S2	S1	S0	U5	U4	U3	U2	U1	U0	A4	A3	A2	A1	A0	N9	N8	N7	N6	N5	N4	N3	N2	N1	N0	Total
Number of pm		1799	1081	1269	0	0	658	63	78	73	113	24	221	67	457	93	501	513	0	120	124	7	30	27	79	34	81		7512
Length	Mean	3310	3354	2848	–	–	2687	2537	2801	3365	3236	2531	3178	2452	2807	2966	2926	3139	–	2034	2136	2158	1738	1343	1946	1656	2035	–	2573
	Median	2753	2614	2162	–	–	1962	2217	2189	2733	2481	2096	2592	1953	2262	2187	2351	2421	–	1540	1801	1769	1594	1233	1541	1399	1365	–	2053
	Min	370	539	24	–	–	415	570	327	302	213	645	126	675	386	506	70	256	–	304	462	882	417	673	436	391	297	–	404
	Max	26,596	22,602	22,767	–	–	14,550	9856	14,650	10,312	13,735	7002	20,911	7774	13,420	15,078	14,944	28,178	–	10,177	7834	3398	4949	2494	9990	5938	16,735	–	13,213
	SD	2296	2536	2405	–	–	2244	1623	2374	2309	2210	1548	2434	1585	1882	2719	2087	2636	–	1464	1392	1042	1024	548	1489	1185	2104	–	1876
	Mean	56	70	63	–	–	66	63	74	62	64	79	56	60	63	64	54	61	–	42	49	44	52	51	53	57	65	–	59
	Median	49	59	57	–	–	53	57	65	58	54	83	51	55	54	63	41	49	–	41	48	40	51	52	50	57	63	–	54
Orientation	Min	0	0	0	–	–	0	0	2	16	0	27	1	13	0	4	0	0	–	0	0	21	14	23	13	19	4	–	7
	Max	180	180	180	–	–	180	177	177	167	176	157	173	167	178	177	179	180	–	179	179	72	96	76	163	90	178	–	159
	SD	36	48	38	–	–	50	46	41	30	41	34	31	26	44	32	43	45	–	28	29	18	19	18	23	16	30	–	33
	RANGE	15	15	21	–	–	17	18	25	27	30	24	20	18	16	22	25	30	–	15	23	27	18	17	25	30	21	–	22
Ploughed surface depth	MEAN	424	633	852	–	–	640	1375	1261	1054	1011	973	722	843	752	991	859	1068	–	740	882	809	645	669	666	823	649	–	841
	STD	4	4	5	–	–	4	5	7	7	8	7	5	4	4	6	6	8	–	4	6	6	4	4	7	8	5	–	6
	Mean	4	4	6	–	–	5	3	4	4	5	4	3	4		3	5	5	–	5	5	6	6	5	6	6	5	–	5
	Median	4	3	5	–	–	5	3	4	4	4	3	3	4		3	5	5	–	5	4	8	5	5	5	6	4	–	4
Keel depth	Min	1	1	1	–	–	1	1	1	1	1	1	1	1		1	1	1	–	1	2	2	1	2	1	1	1	–	1
	Max	14	22	31	–	–	12	10	15	9	17	12	14	9		12	18	30	–	13	12	12	12	13	13	13	17	–	15
	SD	2	3	5	–	–	3	2	3	2	3	3	2	2		2	3	4	–	2	2	4	3	3	3	3	3	–	3
	Mean	210	198	194	–	–	197	208	228	219	250	245	235	157		165	165	226	–	172	206	146	145	157	173	205	171	–	194
	Median	200	175	175	–	–	175	200	200	200	250	225	250	150		150	150	200	–	175	200	150	150	150	175	200	150	–	184
Keel width	Min	100	100	50	–	–	100	50	100	100	100	100	100	75		75	75	100	–	100	150	75	75	75	75	100	100	–	90
	Max	400	600	700	–	–	475	400	450	350	600	450	400	325		325	425	450	–	275	400	200	250	250	300	300	425	–	398
	SD	71	73	48	–	–	51	82	69	73	71	87	93	47		63	38	54	–	44	55	31	33	39	45	46	50	–	57
	Mean	50	60	36	–	–	43	67	50	63	57	80	75	42		50	33	45	–	37	50	23	25	31	32	39	38	–	47
	Median	12	17	8	–	–	11	31	20	17	23	21	25	20		19	7	13	–	14	18	13	15	14	12	15	13	–	16
Keel width/depth	Min	400	350	300	–	–	250	200	200	200	400	200	300	117		200	250	200	–	125	133	57	100	100	200	133	200	–	210
	Max	53	54	38	–	–	36	47	47	38	53	40	56	23		34	25	32	–	21	25	17	21	24	36	28	36	–	36
	SD	53	54	38	–	–	36	47	57	38	53	40	56	23		34	25	32	–		25	17	21	24	36	28	36	–	37

### 4.1 Iceberg ploughmark morphology and dimensions

Overall, ploughmarks are characterized by lengths ranging from hundreds of metres to > 28 km, with average median lengths of ~ 2 km (Table 1). The ploughmark-length frequency distributions for each surface show a typical unimodal character (Fig. 6a) with a positive skew (*i*.*e*., exponential decay in frequency toward longer ploughmarks). Comparing distributions between palaeo-surfaces reveals similar size and geometry of different ploughmark populations (Table 1).

**Fig. 7:**
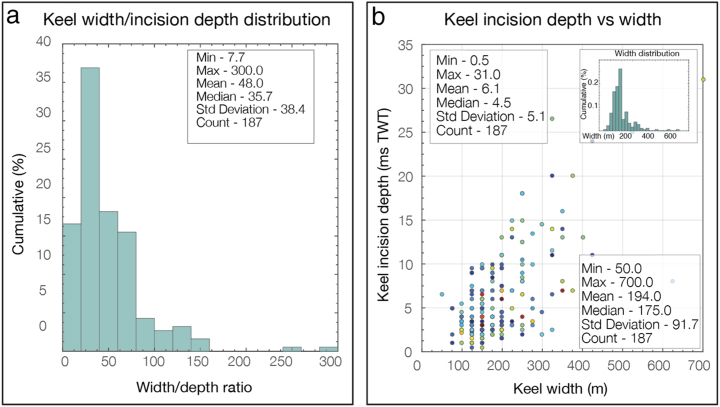
Ploughmark morphology statistics for randomly sampled subsets. (a) Example of frequency distribution of the width to incision depth ratio. (b) Scatter plot of keel incision depth *vs* keel widths with main statistic parameters annotated in the insets. Based on the analysis of random subset sampled from the interpreted ploughmarks within the palaeo-surface T0 (Base of the Sequence T). Data points are coloured according to the ploughmark orientation (see colour key in Fig. 4).

Widths and depths of incisions of the interpreted ploughmarks reach up to 700 m and 30 m, respectively. The frequency-distribution histograms demonstrate a unimodal, positively skewed character (Fig. 6), similar to the length frequency distributions, with average median values of 185 m for width and 4 m for incision depth. Keel depth distributions below the present seafloor, in contrast, show multimodal character (Fig. 6e). Width to depth ratios range from 8 to 400 with an average median of 36:1 (Table 1). In terms of morphology, ploughmarks are most often either u- or v-shaped, with side berms often present although not along every ploughmark. Ploughmarks found within surfaces N tend to be shorter and narrower than within A, U, S and T surfaces (Table 1 and Fig. 7).

**Fig. 8:**
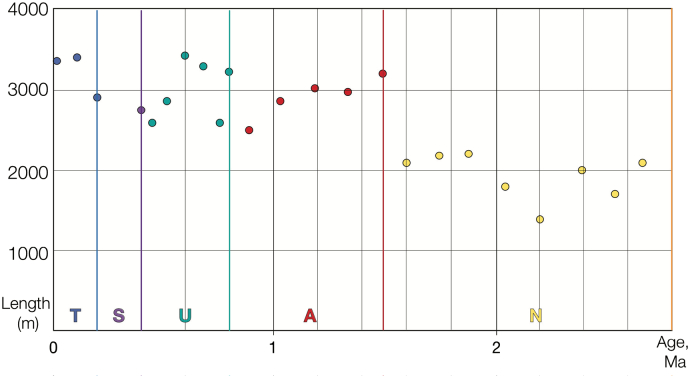
Relationship between palaeo-surfaces and mean lengths of ploughmarks buried within them. Ploughmarks buried within early Quaternary Naust sequence N tend to be considerably shorter than the younger ones.

Overall, there is no clear and consistent relationship between iceberg ploughmark dimensions, their orientations and the mean depth at which they are buried (Fig. 6, Fig. 8). For example, longer ploughmarks do not necessarily tend to have deeper incisions. Similarly, identical direction trends can be observed among ploughmarks that are buried both on the shallow inner shelf and deeper outer slope.

### 4.2 Iceberg ploughmark orientations

**Fig. 9:**
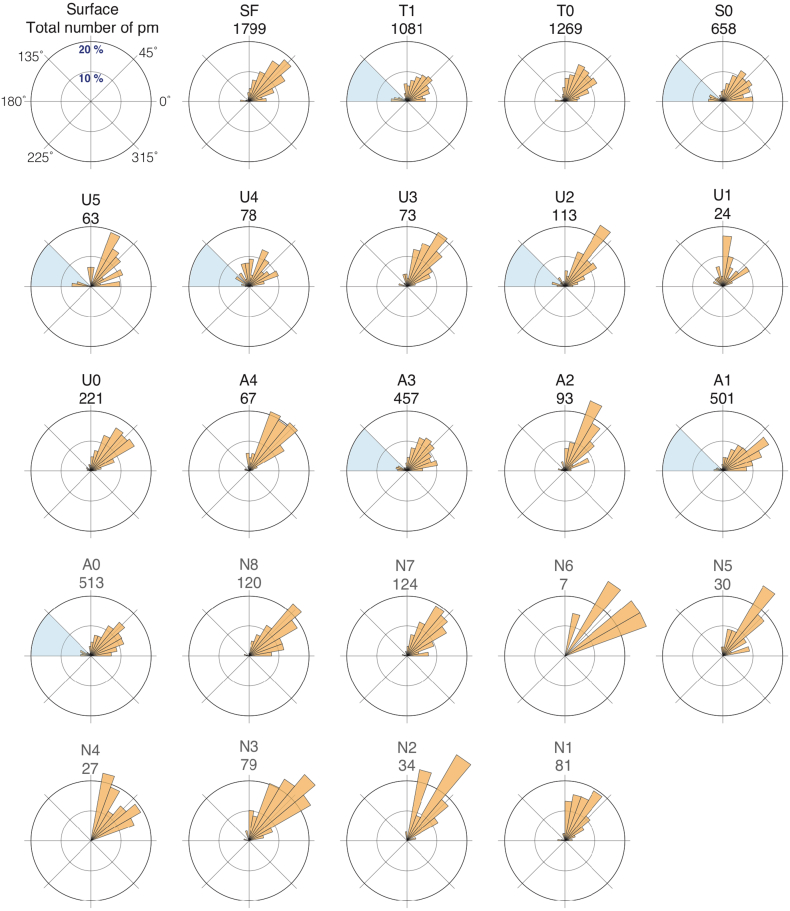
Orientation of iceberg drift in the Norwegian Sea. Rose diagrams showing the normalized frequency distribution of iceberg ploughmark orientations for each of the interpreted Naust palaeo-surfaces. Semi-transparent light blue sectors highlight the surfaces that contain anomalous (*i*.*e*., > 10%) fraction of ploughmarks oriented between 135° and 180°. (For interpretation of the references to colour in this figure legend, the reader is referred to the web version of this article.)

**Fig. 10:**
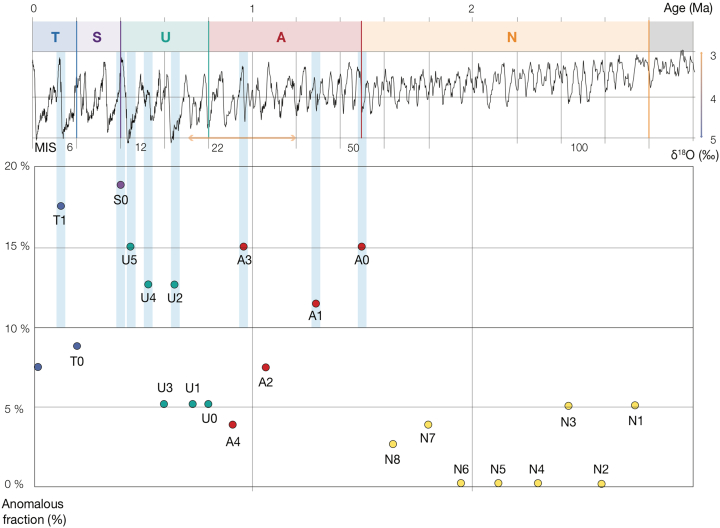
The proportion of buried iceberg ploughmarks orientated between 135° and 180° for each of the interpreted palaeo-surfaces. Vertical axis: percentage of anomalous fraction of ploughmarks (orientated between 135° and 180°). Horizontal axis: tentative timeframe of Naust Formation (Ottesen et al., 2009). Each data point represents an individual palaeo-surface and is positioned according to the provisional age correlations. Semi-transparent light blue bands highlight the surfaces that contain anomalous (*i*.*e*., > 10%) fraction of ploughmarks oriented between 135° and 180°. (For interpretation of the references to colour in this figure legend, the reader is referred to the web version of this article.)

The orientation of iceberg ploughmarks can be determined from their net trajectories and the morphology and direction of terminal grounding pits (Woodworth-Lynas et al., 1991, Hill et al., 2008). Where these small-scale terminal depressions are discernible within 3D seismic data in the study area, their locations suggest a northeastward direction of iceberg drift (Figs. 3a, 5). Thus, the dominant ploughmark orientations are aligned relatively consistently on the interpreted palaeo-surfaces and with modern current directions (Fig. 1, Fig. 9), ranging from 20° to 80° with mean and median orientations across all surfaces at 59° and 54°, respectively (Table 1). Despite the similar trend in major ploughmark directions, there are several palaeo-surfaces (*i*.*e*., T1, S0, U5, U4, U2, A3, A1 and A0) that possess a considerable component (> 10% cumulative) of ploughmarks oriented somewhat differently, at between 135° and 180° (Fig. 9, Fig. 10).

The visual character of orientation-distributions on rose diagrams varies depending on the number of observations. Surfaces that contain more ploughmarks yield relatively smooth, even distributions with less pronounced dominant trends in their direction (Fig. 9). Although all palaeo-surfaces used in this study show largely similar ploughmark orientation trends, care should be taken when interpreting rose diagrams based on small numbers of observations.

## 5 Discussion

The presence of numerous well-developed buried ploughmarks preserved within the Naust Formation implies that drifting icebergs were present on the Norwegian shelf starting from the early Quaternary (Dowdeswell and Ottesen, 2013, Montelli et al., 2017). This has several palaeo-environmental implications.

### 5.1 Palaeo-glaciological implications

#### 5.1.1 Iceberg keel depths and ice sheet thickness

Analysis of iceberg keel depths below the seafloor may provide information on the thickness of the terminus of their parent ice masses (*e*.*g*., Dowdeswell and Bamber, 2007). Our results show that, on the modern seafloor of the mid-Norwegian margin, ploughmarks presumably produced during the last full- and de-glaciation are found at depths ranging from 160 to 900 m, with the majority of them located at depths of 315 ± 50 m (assuming a value of 1500 m/s for seismic velocity in the water column). Even considering that global sea-level was about 120 m lower than today, this demonstrates the occasional presence of extremely thick “megabergs” during the LGM, although most of the ploughmarks are found at much shallower depths – this is consistent with previous observations (*e*.*g*., Jakobsson et al., 2005, Dowdeswell and Bamber, 2007). The identification of a small number of very deep ploughmarks produced by “megabergs” also suggests that maximum ice thickness of the FIS reached values of about 1 km during the calving events that occurred at the LGM and perhaps also during early deglaciation.

Recent observations of icebergs and their source areas in both the Arctic and Antarctic demonstrate that initial iceberg thickness rarely exceeds values of about 600 m and iceberg keels are seldom found on the seafloor at greater water depths (Dowdeswell et al., 1992, Dowdeswell and Bamber, 2007, Dowdeswell and Ottesen, 2013). However, some studies have reported occasional very deep ploughmarks on the continental margins of Greenland, the Canadian and Eurasian Arctic (*e*.*g*., Kristoffersen et al., 2004, Jakobsson et al., 2005, Kuijpers et al., 2007, Metz et al., 2008, Dowdeswell et al., 2010b, Gebhardt et al., 2011, Arndt et al., 2014) and in Antarctica (e.g., Barnes and Lien, 1988). Because the generation of very thick icebergs requires input by fast glacier flow and a calving front close to the grounding line (Stokes and Clark, 2001, Kristoffersen et al., 2004), these deep ploughmarks have been inferred to be produced by large “megabergs” calved at the thick margins of fast-flowing ice streams (*e*.*g*., Vogt et al., 1994, Rignot, 1998, Polyak et al., 2001, Dowdeswell and Ottesen, 2013).

#### 5.1.2 Iceberg source areas

**Fig. 11:**
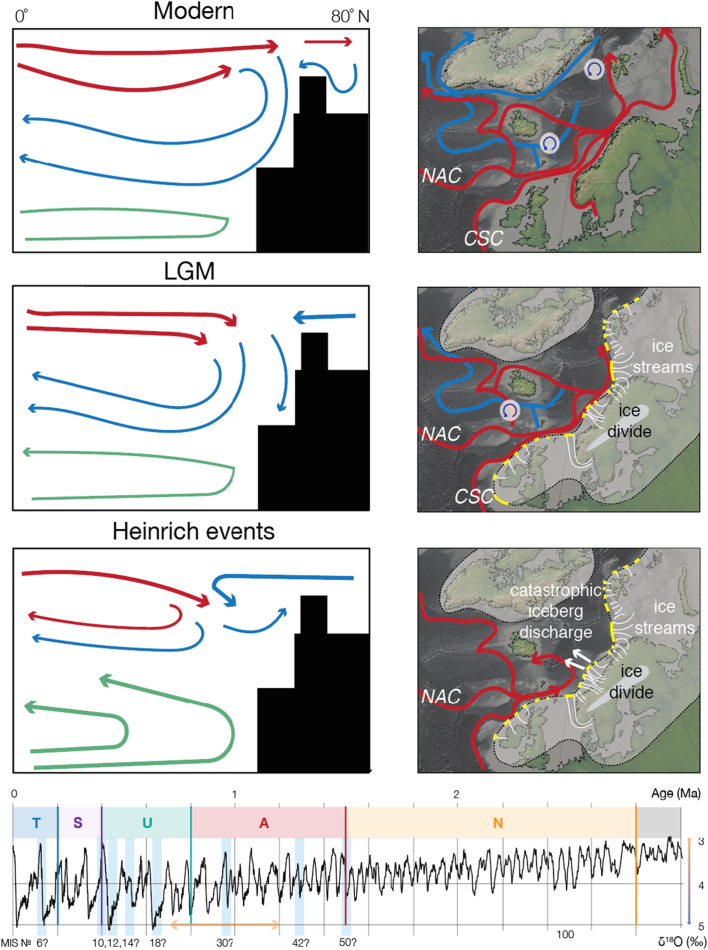
Deep-water overturning circulation in the North Atlantic during modern interglacial, glacial, and Heinrich-event scenarios. Typical overturning patterns are shown in left panel, and corresponding map-view circulation patterns are sketched in the middle panel. Circles with bent arrows show areas of deep-water formation. Modified from Seidov and Maslin (1999). Semi-transparent white areas show previous LGM reconstructions of the Eurasian Ice Sheet and white curved arrows show major LGM palaeo-ice streams that drained the ice sheet (*e*.*g*., Bigg et al., 2012). In the bottom panel: tentative timeframe for the potential NwAC reductions in the Norwegian Sea inferred from the analysis of ploughmark trajectories and the global δ^18^O isotope curve as an ice volume proxy (from Lisiecki and Raymo, 2005).

The wide geographical distribution of iceberg ploughmarks (Fig. 4, Fig. 5) suggests unrestricted iceberg drift and an open Norwegian Sea during the periods of iceberg calving through the Quaternary. Taking into account the location of the study area and previous ice-sheet reconstructions of the Quaternary Eurasian Arctic (Fig. 11), the ice mass predominately producing these icebergs was most likely the FIS that has periodically extended beyond the Norwegian coastline since the early Quaternary (Scourse et al., 2000, Ó Cofaigh and Evans, 2007, Ottesen et al., 2009, Bigg et al., 2010, Dowdeswell and Ottesen, 2013). However, the possibility that some icebergs produced by the British Irish Ice Sheet may have reached the Norwegian Sea *via* the NAC also cannot be excluded. Previous numerical modelling studies have shown that fast-flowing ice streams which drained the FIS were likely the source areas for increased iceberg flux since at least the middle Pleistocene (*e*.*g*., Dowdeswell and Siegert, 1999, Siegert and Dowdeswell, 2002, Dowdeswell and Bamber, 2007, Ottesen et al., 2009).

In this study, we provide iceberg keel depths below the modern seafloor (in ms TWT) for every palaeo-surface within the Naust Formation. However, retrieving the actual palaeo-depths of buried ploughmarks during the time of their formation requires a reliable reconstruction of isostatic fluctuations caused by ice-sheet oscillations and sediment loading in the study area. This, in turn, requires chronologically well-constrained seismic sequences. Although the accurate reconstruction of palaeo-depths is limited because we do not have much of this information, the observation of iceberg ploughmarks on deep parts of the continental palaeo-slope (*i*.*e*., within Sequence U, Table 1) suggests the presence of thick “megabergs” on the mid-Norwegian margin since at least the middle Quaternary. This implies that, by this time, ice streams were already draining the FIS, supporting previous studies that found deeply buried streamlined subglacial landforms indicative of fast ice flow (*e*.*g*., Dowdeswell et al., 2006, Ottesen et al., 2009, Montelli et al., 2017). This is also consistent with previous studies of the Norwegian Channel Ice Stream that has been shown to have initiated around 1.1 Ma–0.8 Ma (Sejrup et al., 1995, Stoker et al., 1983, Ottesen et al., 2014, Ottesen et al., 2016, Batchelor et al., 2017). Given that some parts of ploughed continental slope are deeper than the adjacent continental shelf, these megabergs were probably drifting in from more southerly located parts of the margin that were deep enough for such icebergs to leave the shelf (*e*.*g*., Norwegian Channel Ice Stream and the southern part of the Norwegian Sea).

The distribution of iceberg keel depth values for the palaeo-surfaces in the study area shows a variable, mostly multimodal character, suggesting incursions of different iceberg populations (Fig. 6e). The three peaks in frequency distribution of the iceberg–keel depths, exemplified in Fig. 6e, could be due to calving from different types of iceberg sources: that is, smaller ice shelves calve relatively thin icebergs, whereas deep iceberg keels are generally produced by thick icebergs calved from the margins of fast-flowing outlet glaciers and ice streams (Dowdeswell et al., 1992, Kristoffersen et al., 2004, Dowdeswell and Bamber, 2007). Alternatively, it is also a possibility that large icebergs were initially grounded in deeper waters, where they fragmented into smaller icebergs that could then reach the shallower areas, where further grounding and ploughing occurred (*e*.*g*., Goodman et al., 1980, Sacchetti et al., 2012).

#### 5.1.3 Iceberg ploughmark dimensions

The dimensions of relict iceberg ploughmarks on the mid-Norwegian margin are consistent with previous observations of features from other high-latitude margins (Dowdeswell et al., 1993, Ó Cofaigh et al., 2002, Jakobsson et al., 2005, Dowdeswell and Ottesen, 2013, Arndt et al., 2014). With a mean width of about 200 m and incision depths of up to 30 m, these ploughmarks are similar in dimensions to those previously reported from Arctic and Antarctic seas (Jakobsson et al., 2005, Dove et al., 2014, Arndt et al., 2014) and several times larger than those previously reported from some other areas, including the Labrador Sea (1–2 m deep and 30–40 m wide) (Woodworth-Lynas and Guigné, 1990) and the central North Sea (50–60 m wide) (Dowdeswell and Ottesen, 2013).

The dimensions of icebergs are generally controlled by the thickness and the dynamics of the parent ice sheet and the interactions between the ice margin and marine waters (Dowdeswell and Bamber, 2007). Strikingly different widths and keel incision depths of buried ploughmarks between the two neighbouring areas of the North and Norwegian Seas suggest the presence of larger icebergs in the mid-Norwegian margin compared to the North Sea area. This may indicate a thinner southern margin of the FIS that did not produce relatively large icebergs and/or colder water in the more northerly located Norwegian Sea, which allowed large icebergs to melt more slowly than in the potentially warmer North Sea (*e*.*g*., Dowdeswell and Ottesen, 2013). Another possibility is the presence of bathymetric constraints that prevented icebergs produced at the southern margin of the FIS from drifting into the central North Sea Basin (Dowdeswell and Ottesen, 2013). Previous numerical ice-sheet modelling studies have shown that the Norwegian Channel area represented the southernmost iceberg production zone of the FIS and that it was active for only about 4 kyr around the LGM; a much shorter duration compared to the cross-shelf troughs of the mid-Norwegian continental shelf, where the iceberg production rates were maintained at high values for up to 15 kyr (Siegert and Dowdeswell, 2002). We thus infer that it was likely the combination of ice-sheet thickness, bathymetry and longer duration of iceberg production that explains the larger dimensions of iceberg ploughmarks found in the mid-Norwegian margin compared to the neighbouring North Sea.

Within the early Quaternary sedimentary units of the Naust Formation, preserved iceberg ploughmarks are on average up to half the length of similar features buried within the younger sequences (Fig. 7). This may reflect a combination of several factors, including slower and/or warmer prevailing oceanic currents, fewer icebergs produced in the study area during that period, their smaller dimensions and perhaps also the poorer preservation of the early Quaternary palaeo-surfaces.

Histograms produced for ploughmark dimensions (*i*.*e*., lengths, widths and incision depths) show a positively skewed unimodal frequency distribution (Fig. 6, Fig. 8) similar to previously analysed ploughmarks as well as other glacigenic landforms, such as drumlins or mega-scale glacial lineations (*e*.*g*., Clark et al., 2009, Sacchetti et al., 2012, Spagnolo et al., 2014). This type of metric distribution implies that randomising factors are present in the formation of each of these features (*Fowler et al.,* 2013). This suggests that the long-term formation of multiple generations of iceberg ploughmarks represents an incrementally growing phenomenon where the growth phases occur randomly, or for random durations (*e*.*g*., Wadhams, 1988, Dunlop et al., 2008, Fowler et al., 2013, Spagnolo et al., 2014).

### 5.2 Palaeoceanographic implications

Buried ploughmarks record the history of Quaternary iceberg drift in the study area. Rose diagrams produced for the trajectories of all mapped ploughmarks and the locations of iceberg termination pits (Fig. 3a) demonstrate the strongly prevailing northeasterly orientation of iceberg transport (Fig. 9). This observation suggests that during the numerous calving periods that have occurred on the mid-Norwegian margin through the Quaternary, icebergs were carried by currents flowing in roughly the same net direction as the ocean currents that dominate the modern circulation (Fig. 1, Fig. 9, Fig. 10). Although we infer a largely persistent, northeasterly flowing NwAC during the major iceberg calving events that occurred during the Quaternary, several preserved surfaces do contain a substantial fraction of ploughmarks that exhibit > 90° deviation from their net directional trend (Fig. 9, Fig. 10). This observation suggests potential changes in the current system since ~ 1.5 Ma (Fig. 10) during calving periods that occurred when some of the interpreted palaeo-surfaces (*i*.*e*., T1, S0, U5, U4, U2, A3, A1 and A0) were formed.

Previous ocean circulation models showed that short-lived (*i*.*e*., between 250 and 1250 yr), quasi-periodic ice rafting pulses called Heinrich events could cause the collapse of the deep-water thermohaline conveyor belt in the North Atlantic, shutting down the northern branch of the NAC (*e*.*g*., Heinrich, 1988, Dowdeswell et al., 1995, Andrews, 1998, Seidov and Maslin, 1999, Bigg et al., 2012). Alternatively, such current reductions could be also related to high fluxes of meltwater from retreating margins of ice streams. Additional evidence of centennial-scale NADW reductions that occurred at different points through the Quaternary was provided by epibenthic foraminiferal δ^13^C and δ^18^O records (*e*.*g*., Oppo et al., 1998, Galaasen et al., 2014), as well as in geochemical studies using seawater radiogenic ^231^Nd/^230^Nd and ^231^Pa/^230^Th isotopes (*e*.*g*., Böhm et al., 2015). In addition, a recent 3D seismic investigations by Newton et al. (2016) suggested reduced NAC during Marine Isotope Stage 12 (MIS 12, ~ 430 kyr), based on the ratio between the estimated tidal and geostrophic current velocities inferred from the spiral geometry of buried iceberg ploughmarks of similar geometry to that illustrated in Fig. 3e.

The spatially extensive reconstruction of iceberg trajectories in the mid-Norwegian margin undertaken here shows that an anomalous proportion (*i*.*e*., > 10%) of ploughmarks buried within surfaces S0 and U5 deviated > 90° from the dominant direction trend. This observation may provide additional evidence for a reduced NAC during MIS 12. This reduction has been inferred by Newton et al. (2016) to influence the balance between the tidal currents and the NAC, resulting in the spiral geometry of several iceberg ploughmarks found on ~ 430 kyr old palaeo-surface. In addition, we cannot rule out possible NAC reductions that could have happened, provisionally, around MISs 6, 10, 12, 14,18, 30, 42 and 50 (Fig. 10, Fig. 11), according to the tentatively dated seismic sequences of the Naust Formation (Lisiecki and Raymo, 2005, Ottesen et al., 2009). However, persistent net northeasterly current trends (Fig. 9) imply a relatively short-lived character for these reductions, possibly related to the major iceberg discharges or/and high fluxes of meltwater from ice streams draining the FIS (Fig. 11), particularly given that the majority of anomalous ploughmarks are situated within the deep areas of palaeo-troughs.

Given the critical role of the NAC in the transport of heat from the tropics to polar regions, a scenario in which multiple changes in the configuration of the northern branch of the NAC have occurred over the middle and late Quaternary has important implications for North Atlantic and global climate (*e*.*g*., Rahmstorf, 1995, Clark et al., 2002, McManus et al., 2004, Denton et al., 2010, Boulton et al., 2014). Furthermore, occasional changes of iceberg drift shown in this study suggest a variable spatial distribution of areas where ice-rafted debris was released upon gradual iceberg melting. This phenomenon probably accounts in part for the spatial variability in IRD layers found in the geological record of iceberg-influenced continental margins (*e*.*g*., Dowdeswell et al., 1999).

Geomorphological evidence for ocean current variability on the mid-Norwegian margin provides valuable input for improved reconstructions of the overturning circulation. Unlike sediment cores that represent point-type data, buried ploughmarks may directly elucidate spatial patterns of past ocean circulation. However, some inherent limitations remain in inferring palaeoceanography from the history of iceberg drift using 3D seismic records. First, limited vertical resolution of the seismic data precludes distinguishing between separate generations of iceberg ploughmarks within a single buried palaeo-shelf surface. Therefore, detection of individual iceberg calving events and accurate examination of their timing is problematic. Moreover, smaller-scale features (*e*.*g*., less than ~ 10 m wide and/or deep), including small ploughmarks, side-berms and their detailed morphology may be not distinguishable within the dataset used in this study. Secondly, iceberg ploughmarks can be used as indicators of oceanographic changes only for the periods when a calving ice margin was present on the shelf (*i*.*e*. in and around full-glacial periods), implying the temporally limited nature of such reconstructions. The integration of high-resolution seismic data with more robust chronological control would provide a better insight into the changing nature of iceberg discharge in the mid-Norwegian continental margin through the Quaternary, allowing for more accurate palaeo-environmental reconstructions.

## 6 Conclusions

Multiple 3D seismic cubes covering a vast area (~ 40,000 km^2^) of the mid-Norwegian continental shelf and slope (63–68°N; Fig. 1) were examined for the presence of buried elongate linear and curvilinear incisions. The size and morphology of these features are very similar to those found in other glacier-influenced shelves and slopes, where the seafloor sediments were heavily ploughed by drifting icebergs (e.g. Hill et al., 2008, Dowdeswell and Ottesen, 2013). The features on the mid-Norwegian margin were, therefore, interpreted as iceberg ploughmarks buried within the Quaternary Naust Formation. The ice mass producing the icebergs on the mid-Norwegian margin was probably a FIS that periodically extended across the Norwegian shelf during the past 2.7 Myr.

The morphology and net orientations of > 7500 ploughmarks within 27 palaeo-surfaces preserved in the Quaternary Naust Formation were mapped and analysed. These features are up to 28 km long, with median lengths ranging from 1.2 to 2.7 km for individual palaeo-surfaces. The median width is 185 m and the widest ploughmarks are about 700 m across, generally with berms on either side of a central depression. Ploughmarks are incised up to 31 m into their palaeo-surfaces and are on average 5 m deep. Width to depth ratio ranges from 8:1 to 400:1 with an average median of 36:1. Although rigorous analysis of keel incision water depths is limited to the surface of the modern seafloor, the presence of ploughmarks buried deeply within some palaeo-slope surfaces suggests the occasional presence of very large icebergs, or “megabergs” since the middle Quaternary. This further implies that thick margins of fast-flowing ice streams must have been present in order to calve icebergs of such magnitude into the Norwegian Sea.

Analysis of ploughmark trajectories shows that the ocean-circulation pattern currently dominated by the northeasterly flowing NwAC has persisted throughout the Quaternary, although some palaeo-surfaces contain a considerable fraction of ploughmarks (*i*.*e*., > 10%) that show dramatic, > 90° westerly-orientated deviations from this direction. We interpret this observation as potential evidence for relatively short-lived reductions of the NwAC, possibly related to major pulses of iceberg discharge from the FIS during the middle and late Quaternary, with potential implications for heat transport variability and numerical simulations of global meridional overturning circulation. However, the overall consistent iceberg drift pattern suggests largely persistent NwAC.

This study provides the most extensive margin-wide Quaternary archive of iceberg drift into the Norwegian Sea on glacial-interglacial timescales. Future integration of long, continuous high-resolution sediment cores and seismic datasets available in the study area may develop the most complete history of iceberg transport into the North Atlantic Ocean, providing rigorous constraints for numerical ocean-climate models.
